# Role of long-term memory in object-based attention for the maintenance of binding in visual working memory

**DOI:** 10.3389/fpsyg.2025.1548069

**Published:** 2025-06-09

**Authors:** Nana Sun, Han Han, Peijin Lyu, Ruijun Song

**Affiliations:** ^1^School of Psychology, Shandong Normal University, Jinan, China; ^2^Department of Applied Psychology, Lyuliang University, Lyuliang, China; ^3^School of Psychology, Qufu Normal University, Qufu, China; ^4^Department of Applied Psychology, Changsha Normal University, Changsha, China

**Keywords:** visual working memory, long-term memory, object-based attention, binding, attention

## Abstract

**Background:**

Past research has suggested that binding retention requires more object-based attention than feature retention in visual working memory (VWM). Long-term memory (LTM) is also believed to contribute to VWM.

**Objectives:**

We investigated whether LTM reduces the object-based attention required to maintain bindings.

**Methods:**

Participants were familiarized with specific items prior to the VWM task to establish LTM representations, and we included a Duncan task in the maintenance phase of the VWM task to consume object-based attention.

**Results:**

Results revealed that consuming object-based attention disproportionately impaired bindings compared to features for unfamiliar objects but not for familiar ones (Experiment 1). This effect could not be attributed to differences in memory set sizes between the familiar-objects condition and the unfamiliar-objects condition (Experiment 2) or to differences among participants between the two levels of the LTM condition (Experiment 3).

**Conclusion:**

These findings demonstrate that LTM availability modulates the role of object-based attention in retaining bindings in VWM, with bindings requiring more object-based attention than individual features for unfamiliar objects but not for familiar objects.

## Introduction

Visual working memory (VWM) is a system that has a limited capacity to temporarily store and process visual information to maintain ongoing cognition (Baddeley and Hitch, 1974). A key characteristic of VWM is that it stores objects as collections of features (Hu et al., 2014). The process of binding various features into an integrated object is known as the “binding problem” (Treisman, 1998). Perceptual research has suggested that attention must be paid to the binding of visual features to form a holistic percept (Treisman and Gelade, 1980). However, it is not yet clear whether any attention is required to maintain bindings in the VWM (Allen et al., 2012; Johnson et al., 2008; Shen et al., 2015; Wheeler and Treisman, 2002).

Researchers have spent two decades investigating whether maintaining feature binding in VWM demands more attention than maintaining features. Luck and Vogel (1997) used a change-detection paradigm and found no difference in VWM performance between objects composed of a single feature and those composed of multiple features. Researchers further employed the contralateral delay activity (CDA), an electrophysiological index reflecting VWM capacity, and discovered that the amplitude of the CDA varied with the number of objects, not the number of features contained within the objects (Luria and Vogel, 2011; for reviews, see Luria et al., 2016). These studies suggest that the unit of VWM storage is the object rather than the individual features, implying that features constituting an object are automatically bound in VWM without needing attention. Wheeler and Treisman (2002) used two probe paradigms to examine this issue: whole-probe paradigm and partial-probe paradigm. The test array contained the same number of objects as the memory array in the whole-probe paradigm, while in the partial-probe paradigm, only one object served as the probe. They found that when using the whole-probe method, the memory performance for bindings was poorer than that for features. In contrast, when using the partial-probe method, the memory performance was equivalent under conditions of bindings and features. Wheeler and Treisman (2002) proposed that maintaining feature binding in VWM required attention, but in the whole-probe method, attention was redistributed to other items, leading to the disintegration of the bound representation, which did not occur in the partial-probe method.

Researchers have mostly used the dual-task paradigm in recent years to examine how three different attentional styles—space-based attention, central attention, and object-based attention—affect the retention of feature binding in VWM. In the dual-task paradigm, a set of items was presented to participants, who were instructed to memorize either features or bindings between features. During the retention phase of VWM, a secondary task that consumed specific attentional resources was inserted (for reviews, see Schneegans and Bays, 2018). This would imply that if the secondary task impairs bindings more than features, attentional resources are needed to maintain feature binding in VWM. Using this paradigm, the researchers found that a supplementary task that diverted object-based attention worsened binding performance more than feature performance (He et al., 2020; Shen et al., 2015; Zhou et al., 2021). By adding a supplementary task that consumed either central attention (Allen et al., 2014; Allen et al., 2012; Baddeley et al., 2011) or space-based attention (Johnson et al., 2008; Shen et al., 2015), the impairment did not, however, differ between bindings and features. These findings imply that feature binding maintenance in VWM requires more object-based attention than feature maintenance in contrast to space-based or central attention.

LTM has been shown to improve VWM in extensive empirical work. Researchers have found that more items with LTM representations were retained in VWM than those without. Jackson and Raymond (2008) used a change-detection paradigm to study VWM for unfamiliar faces and famous faces, which were presented upright or inverted. They discovered higher VWM capacity for famous faces than unfamiliar ones, and this effect disappeared when faces were inverted. Nishimura et al. (2022) also found that VWM capacity for own-race faces was higher than that for other-race faces. The conclusion that LTM helps increase VWM capacity can be extended to memory items beyond faces (Curby et al., 2009; Xie and Zhang, 2017) and different-aged participant groups (Starr et al., 2020; Rhodes et al., 2022). After controlling for the confounding factor of visual complexity of memory items, researchers still found that VWM capacity depended on the LTM semantic knowledge of the items (Ngiam et al., 2019; Asp et al., 2021; Conci et al., 2021). The increased VWM capacity for items with LTM representations was due to more items being retained in VWM rather than direct retrieval of these items from LTM during the VWM memory retrieval phase (Brady et al., 2016). Although the above studies show that LTM can enhance VWM, it is unclear how LTM facilitates VWM.

The maintenance of feature binding in VWM demands greater object-based attention than feature retention. Emerging evidence suggests that LTM facilitates VWM. We speculate that this facilitation may occur through the capacity of LTM to reduce the object-based attention required for feature binding in VWM. This hypothesis is grounded in the following evidence: Despite findings in the perceptual field suggesting that feature binding requires attention, a study conducted on visual extinction patients, who had difficulty detecting contralesional stimuli when it was presented simultaneously with a competing ipsilesional item, showed that presenting stimuli with familiar color–shape bindings in the contralesional field reduced extinction (Rappaport et al., 2016). This indicates that LTM can help reduce the attention required for feature binding in perception. Previous studies suggest that the underlying mechanisms of perception and working memory share similarities (Gao and Bentin, 2011; Kiyonaga and Egner, 2014; Mayer et al., 2007). Therefore, it is possible that LTM may have a role in reducing the object-based attention that underlies the rehearsal of feature binding in VWM. LTM may reduce the demand for object-based attention through two potential mechanisms: (1) Pre-stored feature binding in LTM could be automatically retrieved during VWM maintenance to support the maintenance of feature binding (Brady et al., 2016), thereby minimizing the need for object-based attention; (2) Since VWM is a restoration of the activation of LTM (Lewis-Peacock and Postle, 2008), and object-based attention (Peters et al., 2015) and VWM (Xu, 2017) also involve similar neural mechanisms (e.g., parietal lobe), well-established feature binding in LTM (e.g., the association between “red” and “circle” for a canonical stop sign) may generate top-down predictions that pre-activate integrated object representations in VWM. Consequently, when maintaining familiar binding in VWM, the brain can rely on these pre-activated neural templates, thereby reducing the need to allocate object-based attention for the familiar binding.

To elucidate the mechanism of interaction between LTM and VWM, in the current study, we examined whether LTM helps reduce the attention consumed by feature binding in VWM. To meet this aim, the VWM task was preceded by a long-term learning phase in which participants learned six colored shapes. Meanwhile, we tapped object-based attention by adding a Duncan task (Duncan, 1984) to the maintenance phase of VWM, in line with previous studies (Shen et al., 2015; He et al., 2020; Zhou et al., 2021). We predicted that if LTM can help reduce the attention required by feature binding in VWM, then the selective binding impairment that emerges in the unfamiliar objects condition will decrease or vanish for the familiar objects involved in the LTM learning phase. However, if LTM does not modulate the attention consumed by feature binding in VWM, the selective binding impairment will be observed for both familiar and unfamiliar objects in VWM, with a comparable magnitude of impairment.

## Experiment 1: unfamiliar objects are created by randomly combining six different colors and six different shapes

The experiment consisted of two phases. In the first phase, which was the LTM learning phase, participants freely learned six colored shapes. The second phase tested VWM for familiar objects involved in the LTM learning phase and unfamiliar objects with no pre-existing LTM representations. Unfamiliar objects were created by randomly combining six different colors and six different shapes distinct from those of familiar objects. During the VWM maintenance phase, a Duncan task was inserted to consume object-based attention. This task has been widely accepted in demonstrating object-based attention (Chen, 2012; Duncan, 1984; Matsukura and Vecera, 2009). In this task, we presented participants with two superimposed objects: a box and a line, each containing two task-relevant features. Participants were asked to report two features from one object or two distinct objects. We compared the magnitude of the selective binding impairment for familiar and unfamiliar objects.

### Method

#### Participants

The necessary sample size for this investigation was calculated using G*Power 3.1.9.2 (Faul et al., 2009). Repeated-measures analysis of variance (ANOVA) for the within-between interaction effect (2 × 3 × 2) was selected. The sample size was estimated using a moderate effect size of *f* = 0.25, *α* = 0.05, and 1 − *β* = 0.95. According to the computation, each group needed 14 participants. In line with previous related studies that selected 24 participants (Gao et al., 2017; Shen et al., 2015) and to enhance the statistical robustness, the sample included 24 participants (six males, aged between 17 and 20 years, *M* = 19.25, *SD* = 0.74) in the familiar-objects condition and 24 participants (six males, aged between 20 and 21 years, *M* = 20.29, *SD* = 0.46) in the unfamiliar-objects condition. Due to chance-level performance in the Duncan task, three individuals in the familiar objects condition and five participants in the unfamiliar-objects condition were replaced with newly recruited subjects who demonstrated above-chance accuracy in the Duncan task. All participants were undergraduate students from Lyuliang University who filled out consent forms and had normal or corrected-to-normal visual acuity (including color vision). This study was approved by the Ethics Committee of Qufu Normal University.

#### Apparatus and stimuli

##### Stimuli

Participants were seated in a dark room, approximately 60 cm from a screen. Stimuli were presented against a gray (RGB: 128, 128, 128) background on a 19-in. CRT monitor with a resolution of 1,024 × 768 pixels at a 60-Hz refresh rate, controlled by E-prime 2.0 software.

##### LTM learning task

The stimuli for the LTM learning phase comprised six colored shapes: blue chevron, green diamond, magenta circle, red triangle, white cross, and yellow star. The corresponding RGB color codes were as follows: blue (0, 0, 255), green (0, 255, 0), magenta (255, 0, 255), red (255, 0, 0), white (255, 255, 255), yellow (255, 255, 0). Each shape subtended a visual angle of 1.31° × 1.31.

##### WM task

The memory array consisted of three colored shapes. For the condition of the familiar objects, three colored shapes in the memory array for each trial were randomly selected from the six pre-learned stimuli in the LTM learning phase. For the unfamiliar-objects condition, three colored shapes in the memory array per trial were drawn from 36 stimuli generated by pairing six colors (blue, green, magenta, red, white, yellow) with six shapes (chevron, diamond, circle, triangle, cross, star). Probed stimuli differed across conditions: color-only (colored blobs), shape-only (hollow shapes), and binding (colored shapes).

##### Duncan task

**Figure 1:**
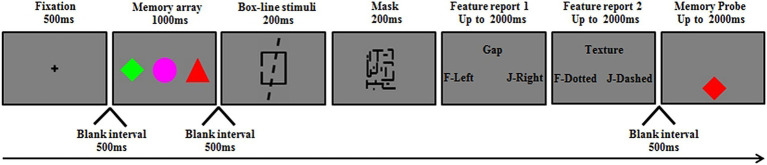
A schematic illustration of a trial used in Experiment 1 VWM task. This figure shows an example of familiar objects with Duncan task-binding condition, and the bindings between features of memory items were changed.

The stimuli in the Duncan task consisted of two superimposed objects: a box and a line (see Figure 1 for an illustration). Each object had two task-relevant features. The box (0.67° width) was either short (0.67°) or tall (1.14°), which had a gap (0.20° width) in the center of either its left or its right side. The line (1.53° long) was either dashed or dotted, and it tilted 8° to either the left or the right. After the superimposed objects, a backward mask (2.10° × 1.62°) was immediately presented.

#### Design and procedure

In experiment 1, we used a 2 (LTM condition: familiar objects vs. unfamiliar objects) × 3 (memory condition: color, shape, color-shape binding) × 2 (second task load: no Duncan task vs. with Duncan task) mixed factorial design. LTM condition served as a between-subject factor. Within the VWM task, familiar objects referred to the memory items studied during the LTM learning phase, and unfamiliar objects referred to the memory items not part of the LTM learning phase. Memory condition and second task load were within-subject factors. The VWM experiment was divided into two sessions based on the second task load. The order of the two sessions was counterbalanced between subjects using an ABBA scheme. Each session consisted of three blocks according to memory condition: color block, shape block, and color-shape binding block, the order of which was fully counterbalanced among participants using a Latin square. Each block consisted of 32 trials, yielding 192 trials per participant. Participants were required to complete 10 practice trials before each block to ensure task comprehension.

The whole experiment comprised two phases. The initial phase, designated as the LTM learning phase, was administered exclusively to participants in the familiar-objects LTM condition. During this phase, participants first engaged in unrestricted visual study of six colored shapes, followed by a test. The test procedure was as follows: First, a black fixation cross appeared in the center of the screen for 800 ms. Then, a colored shape was presented on the screen for 150 ms, either a studied item or a novel color-shape combination. Participants were required to judge whether the colored shape matched the learned set, and if the participants did not press a key within 2,000 ms, the colored shape would disappear. The computer provided feedback on the correctness of every response. To suppress verbal encoding strategies, participants were instructed to continuously rehearse the Chinese characters ‘东南西北’ (dōng nán xī běi, meaning ‘east, south, west, north’) at a normal speaking pace throughout the experiment. The test consisted of 100 trials, with an equal number of “yes” and “no” responses, and lasted approximately 10 min. If the participants achieved an accuracy rate of 0.9 or above in two consecutive tests, they were considered to have preliminarily memorized the six colored shapes. After the test, the electronic versions of the study shapes were sent to the participants, requiring them to study the six shapes for at least 10 min on both the initial and subsequent training days. Participants were also asked to return on the second day to participate in the VWM task. To verify whether the participants had formed LTM for the six shapes, they were asked to take the test again before the VWM experiment. If the accuracy rate was below 0.9, they could not participate in the VWM experiment.

The second phase was the VWM task. All participants completed the VWM task. Each block commenced with instructions specifying the memory condition (color, shape, or color-shape binding) and whether concurrent engagement in the Duncan task was required. A central cross (500 ms) signaled trial onset. Following a 500-ms blank interval, three colored shapes were displayed for 1,000 ms (see Figure 1). Depending on the memory condition, participants were instructed to memorize color, shape, or color-shape binding. After a blank interval of 500 ms, a Duncan task was presented, during which a superimposed box-line stimulus was presented for 200 ms, followed by a mask presented for 200 ms. Then, two successive questions probing the feature dimension of the box-line stimuli were presented sequentially, each lasting a maximum of 2,000 ms. In the no-Duncan task condition, participants were told to disregard the questions by pressing the spacebar on the keyboard to proceed, while in the with-Duncan task condition, participants were required to press the appropriate keys on the keyboard to respond to the two questions. Then, after a 500-ms interval, the memory probe was presented 1.6° below the center of the screen, and participants were told to press the “F” key when the probe was in the memory array within 2,000 ms (50% of trials) or the “J” key when the probe was absent (50% of trials). When the probe was absent from the memory array, a new feature or binding not presented in the memory array appeared. Two different features from two randomly selected objects in the memory array were recombined to form the new binding. Accuracy was prioritized for both VWM and secondary tasks. The maximum delay duration of the VWM task (i.e., the task presentation time when participants do not make any response) was 9,400 ms, which was the same under the two secondary task load conditions.

#### Analysis

Only trials with accurate secondary task responses were retained for further analysis. To have a direct comparison with previous studies on this topic (He et al., 2020; Shen et al., 2015). The performance in the VWM task was reported as corrected recognition (hits-false alarms) (He et al., 2020). A 2 (LTM condition: familiar objects vs. unfamiliar objects) × 3 (memory condition: color, shape, color-shape binding) × 2 (second task load: no Duncan task vs. with Duncan task) repeated measures ANOVA was conducted on the corrected recognition, with memory condition and second task load as within-subject factors, and LTM condition as a between-subject factor. Planned contrasts were conducted to deconstruct the LTM condition × memory condition × second task load interaction. We first calculated the difference between the no-Duncan task and the with-Duncan task condition as the cost. We then ran planned contrasts by separately comparing the cost under the binding condition with the cost under the two single-feature conditions for familiar and unfamiliar objects. In addition, because the accuracy of the secondary task was high and was not our analysis of interest, we did not analyze the accuracy of the secondary task.

### Results

The overall accuracy of the Duncan task was 96.72%. The descriptive data of the VWM task are reported in Table 1.

**Table 1:** Hit rate, false alarm, and correct rejection (CR) under each condition in Experiment 1 (E1), Experiment 2 (E2), and Experiment 3 (E3).

Experiment	LTM condition	Second task load	Color			Shape			Binding		
			Hit	False alarm	CR	Hit	False alarm	CR	Hit	False alarm	CR
E1	Familiar objects	No Duncan task	87.39 (4.19)	10.00 (1.63)	81.52 (2.62)	84.60 (4.15)	16.77 (2.32)	71.95 (3.77)	88.46 (4.09)	6.30 (1.46)	86.28 (2.19)
		With Duncan task	87.02 (1.33)	8.75 (1.77)	78.28 (2.29)	79.94 (4.34)	16.67 (3.35)	67.38 (4.96)	95.99 (1.06)	8.55 (1.84)	87.45 (2.03)
	Unfamiliar objects	No Duncan task	94.48 (1.49)	9.29 (2.41)	85.19 (3.49)	77.25 (3.38)	14.65 (2.78)	62.60 (4.21)	72.61 (3.72)	20.91 (3.50)	51.76 (5.55)
		With Duncan task	91.82 (2.30)	15.47 (2.80)	76.27 (4.13)	72.06 (3.82)	16.63 (2.29)	55.18 (3.98)	58.41 (4.08)	31.01 (3.32)	27.51 (5.72)
E2	Familiar objects	No Duncan task	92.38 (1.51)	11.62 (1.99)	80.76 (2.16)	91.25 (1.90)	15.52 (2.54)	75.73 (3.11)	94.24 (1.77)	8.85 (3.85)	88.96 (2.67)
		With Duncan task	85.27 (1.85)	10.78 (2.23)	74.48 (3.07)	85.42 (2.92)	13.15 (2.28)	72.26 (4.22)	94.28 (1.41)	5.46 (1.39)	88.82 (2.10)
	Unfamiliar objects	No Duncan task	89.95 (3.48)	9.03 (2.21)	80.92 (4.55)	82.69 (3.65)	11.98 (1.83)	70.71 (4.70)	90.47 (2.62)	12.96 (1.81)	77.51 (3.37)
		With Duncan task	84.56 (2.95)	8.11 (1.88)	76.45 (3.58)	78.47 (4.03)	12.21 (2.04)	66.51 (4.55)	77.60 (4.07)	15.78 (2.46)	61.82 (5.12)
E3	Familiar objects	No Duncan task	94.53 (1.47)	6.81 (1.73)	87.92 (2.82)	88.59 (2.22)	10.73 (1.71)	78.00 (2.72)	88.28 (2.48)	8.16 (1.65)	80.25 (3.67)
		With Duncan task	88.39 (2.32)	6.15 (1.55)	82.33 (2.72)	86.71 (2.52)	10.20 (1.84)	75.46 (3.44)	88.92 (2.45)	8.58 (2.38)	80.42 (3.48)
	Unfamiliar objects	No Duncan task	87.96 (1.67)	12.54 (2.25)	75.43 (2.64)	84.29 (2.10)	12.24 (1.99)	72.05 (3.13)	83.82 (3.31)	12.64 (2.27)	71.86 (4.17)
		With Duncan task	89.03 (2.60)	17.84 (2.59)	71.19 (4.39)	83.14 (3.18)	12.66 (2.29)	70.48 (3.82)	73.59 (4.36)	16.48 (2.61)	57.82 (5.07)

The data in parentheses are MSE.

**Figure 2:**
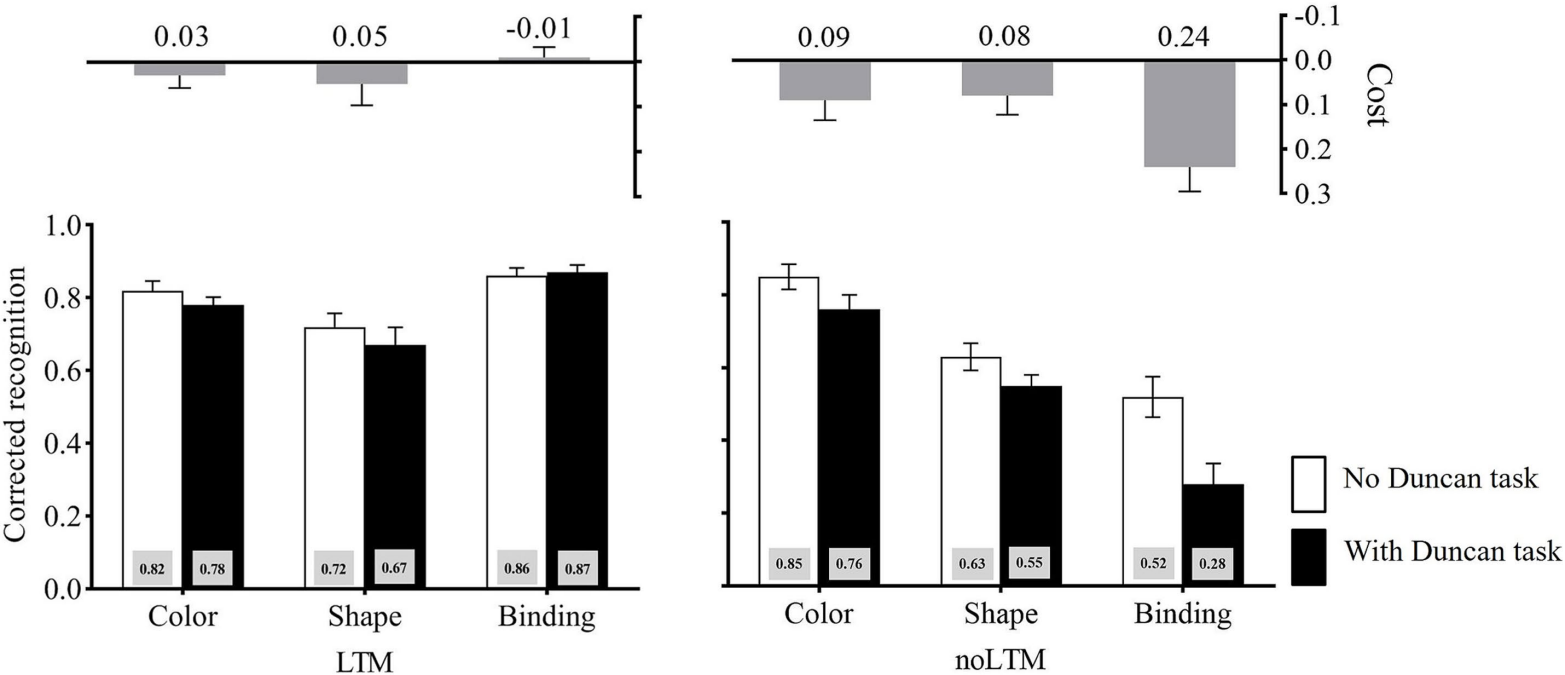
Corrected recognition in Experiment 1 across LTM-condition, memory-condition, and second task load. The cost is calculated as the difference between “no Duncan task” and “with Duncan-task” conditions (no Duncan task minus with Duncan task). Error bars stand for standard errors.

The corrected recognition in each condition is shown in Figure 2. The three-way ANOVA on the corrected recognition revealed a significant main effect of the LTM condition, *F*(1, 46) = 40.59, *p* < 0.001, η_p_^2^ = 0.47, suggesting that the performance was significantly worse for unfamiliar objects (*M* = 0.60) than for familiar objects (*M* = 0.80). The main effect of the second task load was significant, *F*(1, 46) = 19.80, *p* < 0.001, η_p_^2^ = 0.30, showing that the performance was significantly better in the no-Duncan task condition (*M* = 0.74) than in the with-Duncan task condition (*M* = 0.66). The main effect of memory condition also reached significance, *F*(2, 92) = 20.72, *p* < 0.001, η_p_^2^ = 0.31. Further analysis (Bonferroni-corrected) revealed that the performance was significantly higher for color (*M* = 0.81) than for shape (*M* = 0.65, *p* < 0.001) and binding (*M* = 0.64, *p* < 0.001), but there was no significant difference between shape and binding, *p* = 1.000. The interaction between LTM condition and second task load, *F*(1, 46) = 8.15, *p* = 0.006, η_p_^2^ = 0.15, and the interaction between LTM condition and memory condition, *F*(2, 92) = 37.37, *p* < 0.001, η_p_^2^ = 0.45, were significant. Critically, there was a significant LTM condition × memory condition × second task load interaction, *F*(2, 92) = 5.41, *p* = 0.006, η_p_^2^ = 0.11.

Planned contrasts deconstructing the three-way interaction showed that, in the unfamiliar objects condition, the cost caused by the Duncan task was larger under binding (*M* = 0.24) than under color (*M* = 0.09), *t*(23) = 2.62, *p* = 0.015, Cohen’*d* = 0.54, and shape (*M* = 0.08), *t*(23) = 2.59, *p* = 0.016, Cohen’*d* = 0.53. By contrast, in the familiar objects condition, the cost caused by the Duncan task showed no difference between binding (*M* = −0.01) and color (*M* = 0.03), *t*(23) = −1.20, *p* > 0.05, Cohen’*d* = 0.24, and shape (*M* = 0.05), *t*(23) = −1.19, *p* > 0.05, Cohen’*d* = 0.24.

### Discussion

In experiment 1, we investigated whether retaining bindings in VWM required more object-based attention than retaining features for familiar and unfamiliar objects. Participants in the familiar-objects condition were required to form LTM representations of six colored shapes through pre-training prior to the VWM task. On the following day, this group of participants completed the VWM task using these pre-learned stimuli exclusively as their memory set. In contrast, participants in the unfamiliar-objects condition only performed the VWM task with a memory set of 36 objects created by random combinations of six colors and six shapes. A Duncan task consuming object-based attention was inserted into the maintenance phase of the VWM task. Three main findings emerged. First, accuracy was significantly higher in the familiar-objects condition than in the unfamiliar-objects condition, aligning with prior evidence that LTM enhances VWM (Asp et al., 2021; Brady et al., 2016; Conci et al., 2021). Second, performance was inferior in the with-Duncan task condition compared to the no-Duncan task condition, suggesting that maintaining objects in VWM requires object-based attention. This finding replicated earlier findings (He et al., 2020; Shen et al., 2015). Third, and most importantly, for unfamiliar objects, the binding cost induced by the Duncan task exceeded that for individual features. However, the binding cost was equivalent to the single feature cost for familiar objects. These results suggest that the maintenance of binding in VWM requires more object-based attention than constituent features for unfamiliar objects. In contrast, the maintenance of binding in VWM does not require more object-based attention than constituent features for familiar objects. In other words, LTM reduces the amount of object-based attention necessary to sustain VWM bindings.

However, in the familiar-objects condition, memory items were randomly selected from a set of six objects, whereas in the unfamiliar-objects condition, memory items were drawn from a set of 36 objects. Thus, the absence of selective binding impairment for familiar objects might have been confounded by the smaller memory set, which enhanced VWM performance for the binding of familiar objects and rendered them less susceptible to interference from the secondary task. To eliminate this confound, we equated the memory set size between the unfamiliar objects and familiar objects conditions in Experiment 2.

## Experiment 2: unfamiliar objects are randomly selected from six colored shapes

In the condition of unfamiliar objects, the memory set was composed of six colored shapes. However, unlike in the familiar-objects condition, participants were not required to learn these objects prior to the VWM task. After equating memory set sizes across familiar objects and unfamiliar object conditions, we re-examined whether retaining bindings in VWM demands more object-based attention than retaining features for unfamiliar objects but not for familiar objects.

### Method

A total of 24 participants (eight males, aged between 17 and 20 years, *M* = 19.25, *SD* = 0.74) participated in the familiar-objects condition, and 24 participants (eight males aged between 20 and 22 years, *M* = 20.92, *SD* = 0.50) participated in the unfamiliar-objects condition. Five participants in the familiar-objects condition and six participants in the unfamiliar-objects condition were replaced because of chance-level performance in the Duncan task. The other aspects were the same as those used in Experiment 1.

In the unfamiliar-objects condition, the memory set was composed of the same colored shapes as in the familiar-objects condition, which was identical to the memory set used in the familiar-objects condition of Experiment 1. The three colored shapes in the memory array for each trial in the unfamiliar objects condition were randomly selected from the six colored shapes in the memory set.

The other aspects were the same as in Experiment 1.

### Results

The overall accuracy for the Duncan task was 95.75%. The descriptive data of the WM task are presented in Table 1.

**Figure 3:**
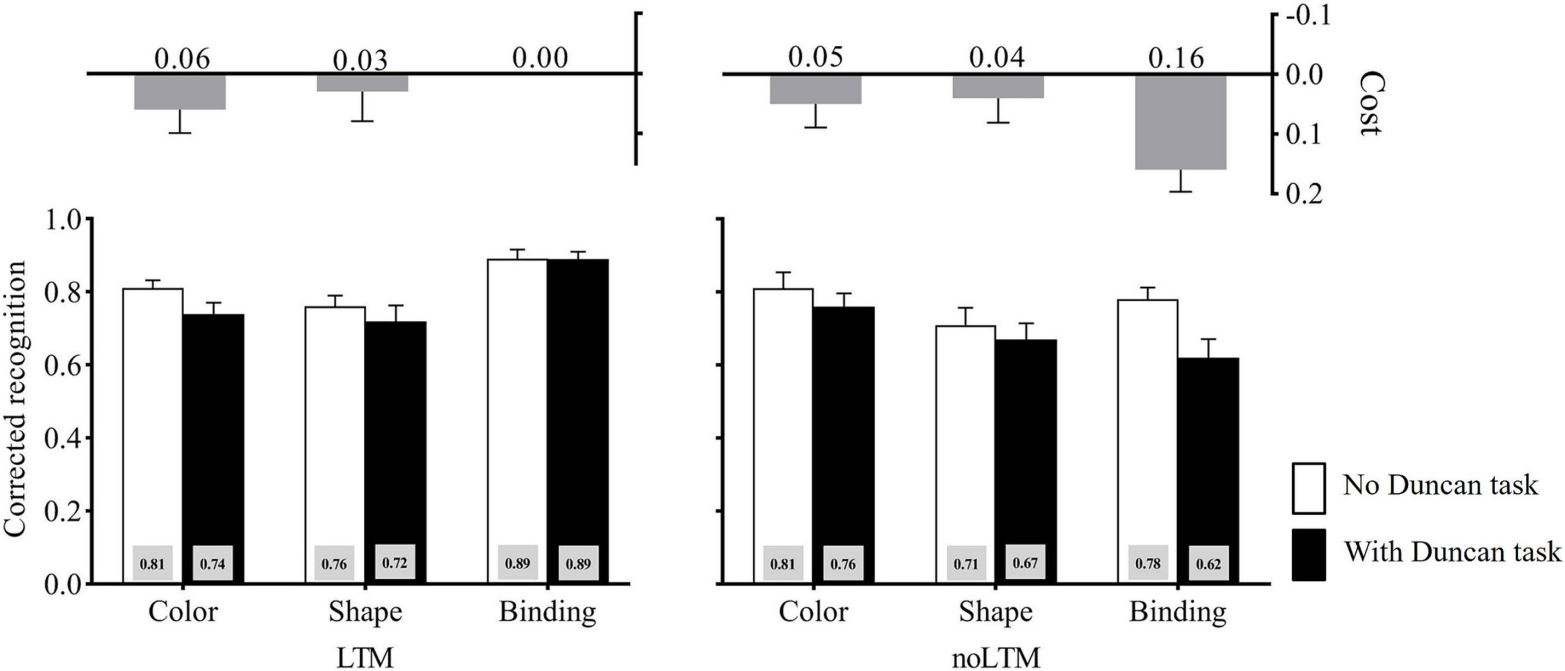
Corrected recognition in Experiment 2 across LTM-condition, memory-condition, and second task load. The cost is calculated as the difference between “no Duncan task” and “with Duncan-task” conditions (no Duncan task minus with Duncan task). Error bars stand for standard errors.

The corrected recognition in each condition is shown in Figure 3. The three-way ANOVA on the corrected recognition revealed a significant main effect of the LTM condition, *F*(1, 46) = 8.10, *p* = 0.007, η_p_^2^ = 0.15, suggesting that the performance was significantly worse for unfamiliar objects (*M* = 0.72) than for familiar objects (*M* = 0.80). The main effect of the second task load was significant, *F*(1, 46) = 10.35, *p* = 0.002, η_p_^2^ = 0.18, showing that the performance was significantly better in the no-Duncan task condition (*M* = 0.79) than in the with-Duncan task condition (*M* = 0.73). The main effect of memory condition also reached significance, *F*(2, 92) = 3.86, *p* = 0.024, η_p_^2^ = 0.077. Further analysis (Bonferroni-corrected) revealed that the performance was significantly higher for binding (*M* = 0.79) than for shape (*M* = 0.72, *p* = 0.017), but the performance of color (*M* = 0.78) was not significantly different from that of shape and binding, *p_1_* = 0.109, *p_2_* = 1.000. The interaction between the LTM and memory conditions was significant, *F*(2, 92) = 5.83, *p* = 0.004, η_p_^2^ = 0.11. Critically, there was a significant LTM condition × memory condition × second task load interaction, *F*(2, 92) = 3.38, *p* = 0.038, η_p_^2^ = 0.069.

Planned contrasts deconstructing the LTM condition × memory condition × second task load interaction showed that, in the unfamiliar objects condition, the cost caused by the Duncan task was larger under binding (*M* = 0.16) than under color (*M* = 0.04), *t*(23) = 2.97, *p* = 0.007, Cohen’*d* = 0.61, and shape (*M* = 0.04), *t*(23) = 2.39, *p* = 0.025, Cohen’*d* = 0.49. By contrast, in the familiar objects condition, the cost caused by the Duncan task showed no difference between binding (*M* = 0.00) and color (*M* = 0.06), *t*(23) = −1.06, *p* > 0.05, Cohen’*d* = 0.22, and shape (*M* = 0.04), *t*(23) = −0.65, *p* > 0.05, Cohen’*d* = 0.13.

### Discussion

Memory performance for feature binding was higher than for shape. This may be due to the fact that for familiar objects, a holistic processing approach was employed (Gauthier et al., 2003; Gauthier and Tarr, 2002), which enhanced memory performance for feature binding.

When memory set sizes were equated between the familiar-objects and unfamiliar-objects conditions in Experiment 2, we still found that the binding cost under the unfamiliar-objects condition remained greater than that for individual features when the Duncan task was imposed. In contrast, for familiar objects, the binding cost under the Duncan task matched that of individual features. These results replicated those of Experiment 1. Both experiments demonstrated that maintaining bindings for unfamiliar objects in VWM demands more object-based attention than constituent features, whereas no such disparity emerged for familiar objects. Together, these findings indicate that LTM attenuates the object-based attentional demands associated with binding maintenance in VWM.

However, in Experiments 1 and 2, the LTM condition was a between-subject variable, leaving unresolved whether individual differences drove outcome disparities between the familiar-objects condition and the unfamiliar-objects condition. To address this confound, the LTM condition was implemented as a within-subject variable in Experiment 3.

## Experiment 3: LTM condition as a within-subject factor

In this experiment, participants completed two VWM tasks sequentially: one involving a memory set of six colored shapes learned during the LTM learning phase and another with six unlearned colored shapes. After controlling for individual differences across the two LTM conditions, we examined whether LTM representations of objects in VWM reduce the demand for object-based attention required to maintain feature binding in VWM.

### Method

#### Participants

For a 2 × 3 × 2 repeated-measures ANOVA with a moderate effect size of *f* = 0.25, *α* = 0.05, and 1-*β* = 0.95, a N = 18 was sufficient to detect an effect with a statistical power of 0.95 for a three-way interaction. A total of 24 participants (six men, aged between 19 and 22 years, *M* = 20.04, *SD* = 0.86) took part in the experiment. Seven participants were replaced due to chance-level performance in the Duncan task. The other aspects were the same as in Experiment 1.

#### Apparatus and stimuli

Stimuli for the LTM learning task comprised six colored shapes: bright-green diamond, bright-red triangle, royal-blue chevron, sky-blue circle, sun-yellow star, and white cross. RGB values were bright-green (0, 255, 0), bright-red (255, 0, 0), royal-blue (65, 105, 225), sky-blue (135, 206, 235), sun-yellow (255, 255, 0), and white (255, 255, 255). Each shape subtended 1.31° × 1.31° of visual angle.

In the unfamiliar-objects condition, three colored shapes in the memory array for each trial were randomly drawn from six stimuli: bright-purple, regular pentagon, coral-pink heart, cyan-green parallelogram, deep-cyan flag, magenta heptagram, and olive-green trapezoid.

The other aspects were the same as in Experiment 1.

#### Design and procedure

The experiment used a 2 (LTM condition: familiar objects vs. unfamiliar objects) × 3 (memory condition: color, shape, color-shape binding) × 2 (second task load: no Duncan task vs. with Duncan task) repeated measures design. All three factors were within-subject factors. In the VWM task, based on the LTM condition, the task was divided into two procedures, the orders of which were counterbalanced between subjects using an ABBA scheme.

The other aspects were the same as in Experiment 1.

#### Analysis

A 2 (LTM condition: familiar objects vs. unfamiliar objects) × 3 (memory condition: color, shape, color-shape binding) × 2 (second task load: no Duncan task vs. with Duncan task) repeated measures ANOVA was performed on corrected recognition scores, with all three factors manipulated as within-subject variables. The other aspects were the same as in Experiment 1.

### Results

The overall accuracy for the Duncan task was 97.37%. The descriptive data of the WM task are listed in Table 1.

**Figure 4:**
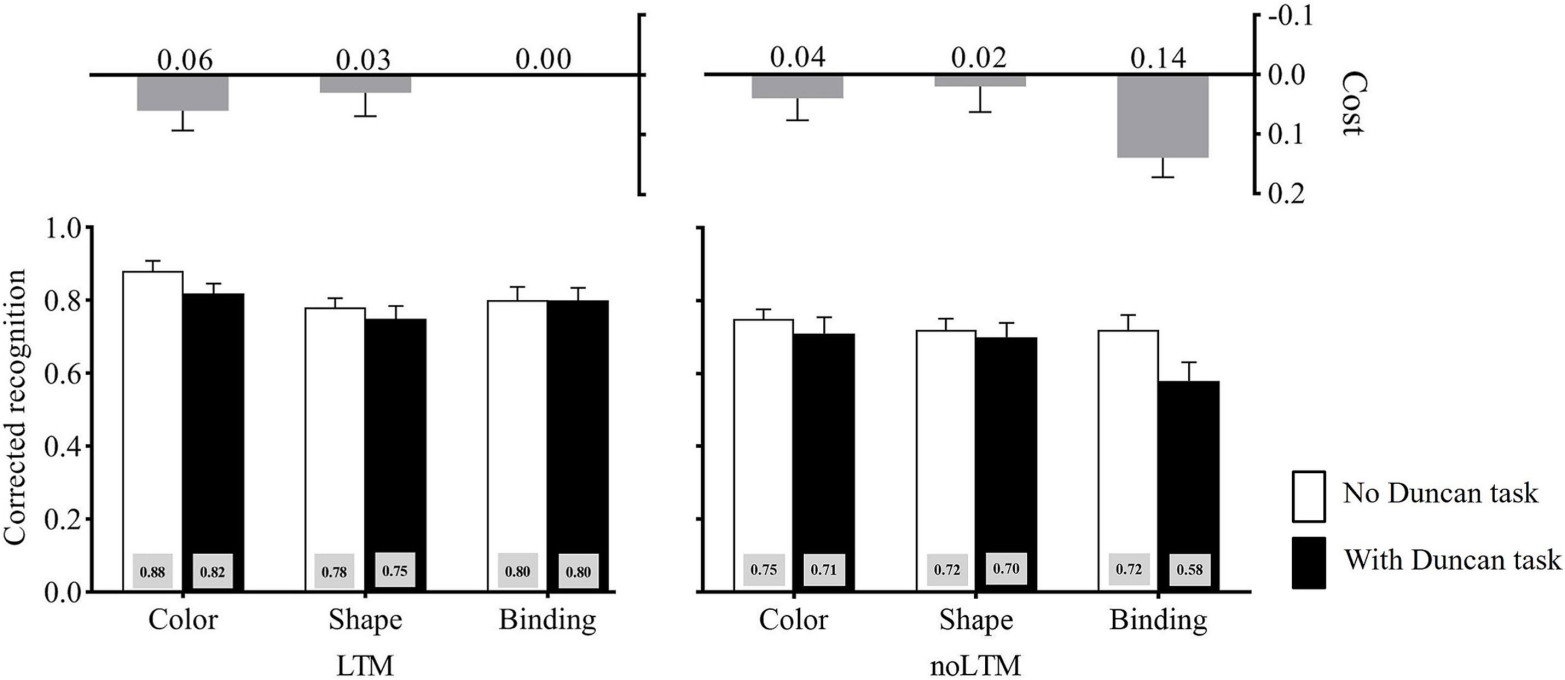
Corrected recognition in Experiment 3 across LTM-condition, memory-condition, and second task load. The cost is calculated as the difference between “no Duncan task” and “with Duncan-task” conditions (no Duncan task minus with Duncan task). Error bars stand for standard errors.

The corrected recognition in each condition is shown in Figure 4. The three-way ANOVA on the corrected recognition revealed a significant main effect of the LTM condition, *F*(1, 23) = 21.69, *p <* 0.001, η_p_^2^ = 0.49, suggesting that the performance was significantly worse for unfamiliar objects (*M* = 0.70) than for familiar objects (*M* = 0.81). The main effect of the second task load was significant, *F*(1, 23) = 6.82, *p* = 0.016, η_p_^2^ = 0.23, showing that the performance was significantly better in the no-Duncan task condition (*M* = 0.77) than in the with-Duncan task condition (*M* = 0.74). The main effect of memory condition also reached significance, *F*(2, 46) = 4.79, *p* = 0.013, η_p_^2^ = 0.17. Further analysis (Bonferroni-corrected) revealed that the performance was significantly higher for color (*M* = 0.79) than for binding (*M* = 0.71, *p* = 0.048), but the performance of shape (*M* = 0.76) was not significantly different from that of color and binding, *p*_1_ = 1.000, *p*_2_ = 0.082. Critically, there was a significant LTM condition × memory condition × second task load interaction, *F*(2, 46) = 3.58, *p* = 0.036, η_p_^2^ = 0.14.

Planned contrasts deconstructing the LTM condition × memory condition × second task load interaction showed that, in the unfamiliar-objects condition, the cost caused by the Duncan task was larger under binding (*M* = 0.14) than under color (*M* = 0.04), *t*(23) = 2.09, *p* = 0.048, Cohen’*d* = 0.43, and shape (*M* = 0.02), *t*(23) = 2.41, *p* = 0.024, Cohen’*d* = 0.49. By contrast, in the familiar-objects condition, the cost caused by the Duncan task showed no difference between binding (*M* = 0.00) and color (*M* = 0.06), *t*(23) = −1.28, *p* > 0.05, Cohen’*d* = 0.26, and shape (*M* = 0.03), *t*(23) = −0.65, *p* > 0.05, Cohen’*d* = 0.13.

### Discussion

In Experiment 3, by implementing the LTM condition as a within-subject factor, results mirrored those of Experiments 1 and 2. We also equated memory set sizes between the familiar-objects condition and the unfamiliar-objects condition, replicating the findings of Experiment 2. After controlling for individual differences and memory set sizes across the two LTM conditions, the binding cost under the Duncan task in the unfamiliar objects condition exceeded that for individual features. Conversely, in the conditions of familiar objects, the binding cost under the Duncan task matched that for individual features. These findings reaffirm that maintaining bindings for unfamiliar objects demands greater object-based attention than individual features, while no such difference exists for familiar objects. This supports the conclusion that LTM mitigates object-based attentional demands during VWM binding maintenance.

## General discussion

The current study examined whether the role of object-based attention in retaining bindings in VWM is modulated by LTM representations of bindings. Participants were trained on specific objects to establish LTM presentations for these objects prior to the VWM task, during which they memorized both familiar and unfamiliar objects while performing a secondary task designed to engage object-based attention during the maintenance phase. Across three experiments, binding retention for unfamiliar objects was disproportionately disrupted by the secondary task compared to constituent features, whereas binding and feature retention for familiar objects were equally impaired. These effects were robust to variations in memory set sizes and individual differences between different LTM conditions. Our findings demonstrate that VWM binding maintenance for unfamiliar objects demands greater object-based attention than feature retention, while no such disparity exists for familiar objects. Thus, the role of object-based attention in binding retention depends on LTM availability for the memorized objects.

The current study contributes to accumulating evidence suggesting that LTM contributes to VWM. Previous research demonstrates that LTM enhances VWM capacity: compared to objects without LTM representations, more objects with LTM representations can be maintained in VWM (Asp et al., 2021; Conci et al., 2021; Jackson and Raymond, 2008; Ngiam et al., 2019; Nishimura et al., 2022). Our findings extend this work by revealing how LTM interacts with VWM, specifically demonstrating that LTM facilitates retaining feature binding in VWM. The underlying mechanism of this facilitation is that LTM attenuates the object-based attention required to maintain feature binding in VWM. For novel objects lacking LTM representations, binding maintenance in VWM requires greater object-based attention than individual features (Shen et al., 2015; Zhou et al., 2021), resulting in fewer retained bindings in VWM. In contrast, for objects with LTM representations, maintaining bindings in VWM does not require additional object-based attention than individual features (as found in the current study), thereby freeing object-based attention to maintain more bindings in VWM, enhancing VWM capacity.

Furthermore, our findings provide insight into the role played by object-based attention in the maintenance of feature binding in VWM, extending our understanding of the scope of the object-based attention hypothesis. Research in perception suggests that LTM attenuates attentional demands for feature binding for familiar objects (Rappaport et al., 2016). We propose that LTM similarly attenuates attentional demands for maintaining feature binding in VWM, such that maintaining feature binding for unfamiliar objects demands greater object-based attention than feature retention. In contrast, no such attentional disparity exists for familiar objects. These results have significant implications for the episodic buffer. Baddeley (2000) added the episodic buffer to his multicomponent working memory model, positing that, supported by central attention from the central executive, the episodic buffer integrates information originating from domain-specific buffers (Baddeley, 2000). However, subsequent studies revealed that maintaining bindings in VWM does not require more central attention than features (Allen et al., 2006; Allen et al., 2012; for reviews, see Baddeley et al., 2011). These results compelled Baddeley and his colleagues to reject their initial hypothesis, instead considering the episodic buffer to be a device that passively stores bindings which are formed elsewhere prior to their integration into the buffer (Baddeley et al., 2011).

Nevertheless, Shen and colleagues found that binding maintenance in VWM demanded greater object-based attention than feature retention (Shen et al., 2015; Zhou et al., 2021). Given that central attention is domain-general (Baddeley, 2000) while object-based attention is domain-specific (Shen et al., 2015). Shen et al. hypothesized that the central executive might involve not only domain-general attention but also domain-specific attention and that the episodic buffer actively stores bindings via object-based attention rather than central attention (Shen et al., 2015; Zhou et al., 2021). Our findings suggest that the function of the episodic buffer depends on whether bindings have corresponding LTM representations: unfamiliar bindings rely on object-based attention for active maintenance, while familiar bindings are stored passively. This aligns with the results presented by Che et al. (2019), which showed that similarity modulates object-based attention for binding retention. While object-based attention was more essential for maintaining dissimilar bindings than constituent features, similar bindings did not necessitate additional object-based attention compared to features (Che et al., 2019). The results of Che et al. (2019) suggest that the episodic buffer requires object-based attention to actively sustain dissimilar bindings but passively maintain similar bindings without the need for attention. In the future, the construction of the theoretical model for the episodic buffer must consider these modulating factors.

The findings of our study hold implications for working memory models. Different models propose distinct relationships between working memory and LTM. In the multicomponent working memory model (Baddeley, 2012), LTM and working memory are structurally separate systems that rely on different representations (Baddeley, 2012). In contrast, the embedded process (Cowan, 1988, 1999) and concentric models (Oberauer, 2002) of working memory suggest that working memory and LTM rely on the same representations, with differences in the level of activation (Cowan, 1988, 1999; Oberauer, 2002). While these models acknowledge interactions between LTM and working memory, they lack specificity regarding the mechanisms of such interactions (Baddeley, 2012; Cowan, 1988, 1999; Oberauer, 2002). Our findings reveal that LTM facilitates working memory by reducing the object-based attention required to maintain feature binding. This enriches and expands theoretical accounts of LTM and working memory interactions within these frameworks.

Why the need for object-based attention in maintaining bindings in VWM is impacted by LTM? The integrated competition model (Duncan et al., 1997) posits that for a multi-feature object when one of the features of the object is attended to, enhanced cortical activity is generated in response, which is then transmitted to cortical regions processing other features of the same object. The competition among objects within the cerebral cortex is integrated across varying cortical regions that are responsible for processing the features constituting the objects. In addition, once a particular feature of an object has been attended to, the remaining features composing the object will dominate in their respective brain regions, leading to an integrated experience of the object (Duncan et al., 1997). Sahan et al. (2020) also discovered object-based selection mechanisms in working memory using neuroimaging techniques. Integrating the perspectives of the integrated competition model with the findings of our study, it can be inferred that LTM may influence the strength of neural activity transmission between the cortical modules that process the features composing an object. Compared to unfamiliar objects, this transmission may be stronger and less interference-prone for familiar objects. Thus, the bindings of familiar objects are not disproportionately affected by secondary tasks that consume object-based attention compared to constituent features.

Alternatively, our findings can be explained through re-entrant processing. In perception, features need to be correctly bound into objects. Re-entrant processing is a top-down processing stream from higher visual cortices (e.g., parietal lobes) to earlier visual cortices, as proposed by feature integration theory (Bouvier and Treisman, 2010). After forming feature binding, re-entrant processing is necessary to check whether the binding is correct based on bottom-up signals, which consumes object-based attention (Bouvier and Treisman, 2010). Since visual perception and VWM share similar processing mechanisms (Gao and Bentin, 2011; Kiyonaga and Egner, 2014; Mayer et al., 2007), VWM binding maintenance may similarly require re-entrant processing. Drawing on the re-entrant processing framework combined with our findings, we propose that LTM modulates re-entrant processing demands during feature binding maintenance in VWM. Depending on LTM availability for the object, the role of re-entrant processing in binding maintenance varies: binding maintenance for unfamiliar objects necessitates re-entrant processing, whereas familiar object bindings and their constituent features do not. Consequently, the secondary task that consumes object-based attention causes greater impairment to the feature binding of unfamiliar objects compared to the constituent features, whereas no such differential impairment occurs for the binding of familiar objects.

LTM may enhance transmission stability between cortical modules processing features of familiar objects and reduce reliance on re-entrant processing to verify binding accuracy for two key reasons. First, participants may consolidate features of familiar objects into cohesive chunks (Cowan, 2001). Chunking involves binding low-level features into strongly associated units that are weakly linked to other chunks (Cowan, 2001). The strong intra-chunk associations allow activity in one cortical module encoding a feature to automatically propagate to other cortical modules encoding other features within the same chunk, stabilizing signal transmission. Second, a chunk actively differentiates the features within a chunk from those of competing objects, enhancing their distinctiveness (Cowan, 2001). Thus, re-entrant processing becomes redundant for verifying binding accuracy.

## Conclusion

Across three experiments, we found that imposing a secondary task consuming object-based attention during VWM maintenance disproportionately disrupted the retention of bindings for unfamiliar objects more than it did for individual features. In contrast, for familiar objects, the task equally impaired bindings and features to the same extent. We conclude that LTM reduces the object-based attentional demands needed to maintain feature bindings in VWM.

## Data Availability

The raw data supporting the conclusions of this article will be made available by the authors, without undue reservation.
